# High-precision and lightweight small-target detection algorithm for low-cost edge intelligence

**DOI:** 10.1038/s41598-024-75243-1

**Published:** 2024-10-09

**Authors:** Linsong Xiao, Wenzao Li, Sai Yao, Hantao Liu, Dehao Ren

**Affiliations:** 1 School of Communication Engineering, Chengdu University of Information Technology, Chengdu, 610225 Sichuan China; 2 Educational Informationization and Big Data Center, Education Department of Sichuan Province, Chengdu, 610015 Sichuan China

**Keywords:** Computer science, Information technology

## Abstract

The proliferation of edge devices driven by advancements in Internet of Things (IoT) technology has intensified the challenge of achieving high-precision small target detection, as it demands extensive computational resources. This amplifies the conflict between the need for precise detection and the requirement for cost-efficiency across numerous edge devices. To solve this problem, this paper introduces an enhanced target detection algorithm, MSGD-YOLO, built upon YOLOv8. The Faster Implementation of CSP Bottleneck with 2 convolutions (C2f) module is enhanced through the integration of the Ghost module and dynamic convolution, resulting in a more lightweight architecture while enhancing feature generation. Additionally, Spatial Pyramid Pooling with Enhanced Local Attention Network (SPPELAN) replaces Spatial Pyramid Pooling Fast (SPPF) to expand the receptive field, optimizing multi-level feature aggregation for improved performance. Furthermore, a novel Multi-Scale Ghost Convolution (MSGConv) and Multi-Scale Generalized Feature Pyramid Network (MSGPFN) are introduced to enhance feature fusion and integrate multi-scale information. Finally, four optimized dynamic convolutional detection heads are employed to capture target features more accurately and improve small target detection precision. Evaluation on the VisDrone2019 dataset shows that compared with YOLOv8-n, MSGD-YOLO improves mAP@50 and mAP@50–95 by 14.1% and 11.2%, respectively. In addition, the model not only achieves a 16.1% reduction in parameters but also attains a processing speed of 24.6 Frames Per Second (FPS) on embedded devices, thereby fulfilling real-time detection requirements.

## Introduction

With the emergence of numerous IoT applications, the deployment of edge-embedded devices has become increasingly prevalent. This trend is particularly prominent in intelligent surveillance systems, where a range of target detection methods have been adopted. Currently, deep learning-based target detection algorithms are generally divided into two categories based on their process characteristics^1^. The first category encompasses two-stage object detection algorithms, primarily exemplified by the Region-based Convolutional Neural Networks (R-CNN) family^2^. While these algorithms achieve high detection accuracy, they are unsuitable for edge-embedded devices due to their high computational demands. The second category comprises single-stage object detection algorithms, such as the Single Shot Multi-Box Detector (SSD)^3^, You Only Look Once (YOLO)^4^, and anchorless models^5^. While the accuracy of these algorithms is generally inferior to that of two-stage methods, their fast detection speed renders them suitable for deployment on edge-embedded devices with limited computational resources. In urban environments, both detection approaches often perform poorly due to the prevalence of small target objects. Small object recognition is particularly challenging due to low resolution and occlusion. Therefore, achieving high-accuracy recognition of small target objects on edge-embedded devices while minimizing deployment and operating costs continues to pose a major challenge. Ensuring the efficient operation of target detection algorithms on low-computation-power edge devices while maintaining high recognition precision remains a primary research focus. Convolutional Neural Networks (CNN) have significantly advanced target detection, with the YOLO series widely adopted for real-time performance^6^. However, as performance requirements for small target detection rise, the number of network parameters also increases. Since YOLO predicts both bounding boxes and categories using a single network with multiple convolutional layers, the computational load has increased significantly.

Numerous researchers have developed diverse algorithms built upon the YOLO network to improve small object detection precision while maintaining real-time performance on low-computation-power devices. Guo et al^7^. proposed a lightweight algorithm, CS-YOLO, that enhances small-target detection precision at the cost of FPS, rendering it unsuitable for edge devices. Likewise, Zhao et al^8^. developed an algorithm that boosts inference speed but remains inadequate in complex scenes. The BD-YOLO algorithm by Lou et al^9^. faces a similar issue, offering marginal improvements in mAP while retaining real-time performance. These approaches fail to strike an effective balance between detection precision and operational cost. Therefore, constructing a target detection model that substantially reduces operational costs while improving small target detection precision remains a valuable task.

The development of small target detection algorithms on edge-embedded devices currently faces two main challenges: one is improving small target detection precision, and the other is reducing operational cost without compromising precision. Therefore, finding an effective method to enhance overall performance is a complex process. To tackle these challenges, this paper proposes the MSGD-YOLO algorithm. The main contributions of this paper are as follows:

1. Building upon the original YOLOv8-n backbone network, a new Ghost Dynamic-C2f (GD-C2f) module replaces the original C2f module, leading to more comprehensive information retention, significantly reducing the number of parameters, and steadily enhancing detection precision. Moreover, SPPELAN^10^ replaces SPPF to capture finer details of small targets.
2. The MSGConv and MSGFPN structures are introduced to efficiently fuse high-level semantic information with low-level details. Additionally, MSGFPN effectively integrates feature map information across different scales while reducing the number of parameters in the model.
3. In the network’s head structure, the Dynamic Head (DyHead)^11^ is employed to replace the original detection head, enhancing feature adaptability and representational capability.
4. Ablation experiments were performed on the VisDrone2019 dataset^12^, and comparative experiments on the neck structure were carried out to demonstrate the method’s effectiveness. On this dataset, our model demonstrated optimal performance compared to the baseline and other mainstream small target detection models.

## Related work

### Small target detection using CNNs

Small target detection, a key area of research in machine vision, aims to accurately locate and identify small objects using specialized algorithms. However, CNN-based target detection algorithms continue to face challenges in achieving high precision, particularly due to low resolution and scale variation in small targets. Researchers have focused on six key strategies to improve small target detection performance:

1. Data enhancement: for instance, Kisantal et al.^13^, Bochkovskiy et al.^14^, and Cubuk et al.^15^ utilize techniques such as copying and pasting small targets in data images or adding additional image data to enhance the model’s robustness, addressing issues related to the subtle features and limited information of small targets, thereby improving detection performance. However, poorly designed enhancement strategies may introduce noise, impair feature extraction performance, and pose challenges for algorithm design.
2. Multi-scale feature fusion: works such as Xu et al.^16^, Ghiasi et al.^17^, and Luo et al.^18^ leverage high resolution at the bottom of the network and strong semantic feature information at the top to enhance the detection accuracy of small targets. However, during the process of multiscale feature fusion, issues such as semantic gaps and noise can arise.
3. Super-resolution techniques: studies by Radford et al.^19^, Li et al.^20^, and Bai et al.^21^ address the low resolution of small targets by employing feature mapping, learning high-resolution feature representations of small targets, and other methods to reduce the feature disparity between small-scale and large-scale targets, thereby improving detection accuracy. However, the Generative Adversarial Network (GAN)^22^ models used can be difficult to train and unstable.
4. Contextual information learning: research by Zhu et al.^23^, Xue et al.^24^, and Qiao et al.^25^ enrich the expression of target feature information in images by leveraging the connections between detected targets and their surrounding objects or environments, thereby enhancing the detection of small targets. However, small objects have fewer pixels, and the weak correlation between objects and their surrounding environment can complicate context learning.
5. Anchor-Free mechanisms: methods proposed by Fu et al.^26^, Qiao et al.^25^, and Ge et al.^27^ design strategies conducive to the detection of small targets through reasonable anchor-free approaches, improving detection accuracy to some extent. Nevertheless, these methods can cause imbalances between positive and negative samples and semantic ambiguity, leading to unstable detection results.
6. Attention mechanisms: techniques developed by Xu et al.^28^, Vajgl et al.^29^, and Zheng et al.^30^ allocate resources efficiently to address the susceptibility of small targets in complex scenes to background interference. These techniques quickly identify regions of interest and ignore distracting information, helping the model obtain global spatial information from feature maps and enriching their contextual semantic information. However, this increases the computational cost of the network model and can affect its ability to extract target features.

These studies demonstrate that each approach to improving small target detection has inherent limitations. To overcome these challenges, it is essential to combine multiple techniques and design tailored network structures for specific scenarios.

### Lightweight network structure

With the ongoing advancements in target detection technology, algorithms have shifted from traditional methods based on handcrafted features to those utilizing deep neural networks. However, current CNN-based detection algorithms demand significant computational resources and memory, leading to higher costs^31^. In contrast, lightweight networks have attracted considerable attention for their ability to trade slight accuracy losses for substantial improvements in detection speed.

Neural network compression techniques can be broadly categorized into four main approaches: parameter pruning and quantisation^32^, low-rank decomposition^33^, lightweight module design^34^, and knowledge distillation^35^. Many researchers have developed various lightweight methods for neural network models. Mardieva et al^36^. designed a lightweight and efficient super-resolution model, optimised specifically for IoT devices. They introduced a Deep Residual Feature Distillation Block (DRFDB), using a Depth-wise Separable Convolution Block (DCB) for efficient feature extraction. This design reduces computational and memory demands while maintaining high image quality, supporting object detection tasks on embedded devices. Wang et al^37^. used YOLOv4 as the framework, incorporating EfficientNet^38^for feature extraction and introducing the lightweight Efficient Channel Attention (ECA)^39^mechanism to mitigate the adverse effects of Path Aggregation Network (PANet)^40^in multi-scale feature fusion. Chen et al^41^. proposed a structural innovation based on the YOLOv5-MobileNetv3-Small model, integrating the MobileNetv3^42^architecture and enhancing the backbone. Tests showed that the improved YOLO algorithm reduced network memory usage by 72.4%. Shen et al^43^. proposed a lightweight convolutional neural network, L-Net, designed for low-computing devices. It achieves a lightweight structure by introducing a Residual Enhanced Channel Attention (R-ECA) module and replacing the traditional Rectified Linear Unit (ReLU) with an Exponential Linear Unit (ELU). Compared to models like MobileNet on image datasets, L-Net achieves higher accuracy and faster operation speed. Hu et al^44^. replaced the convolutional layers in YOLOv3-Tiny with Depth-wise Separable Convolution (DSConv)^45^ and Mobile inverted Bottleneck Convolution (MBConv) and developed a progressive channel-level pruning algorithm to minimize parameters and maximize detection performance, resulting in Micro-YOLO based on YOLOv3-Tiny. While these studies have improved detection speed and reduced parameters for lightweight models in embedded devices, the detection accuracy in real-world applications still requires improvement. Compared to existing methods, this study balances the trade-off between accuracy and operational cost in small target detection, offering a more optimised approach.

## Proposed network

### Overview of the proposed network

**Fig. 1:**
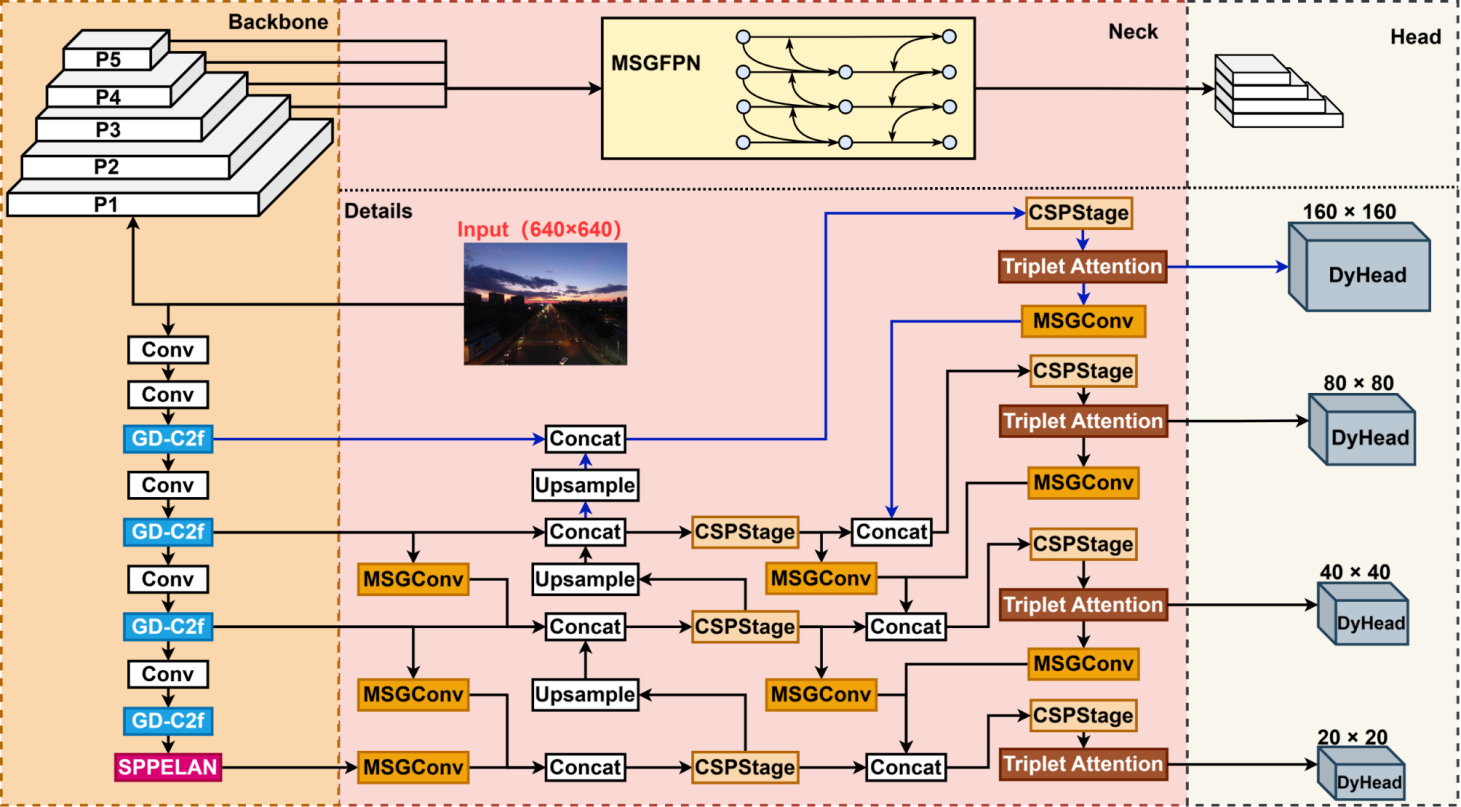
The network structure of MSGD-YOLO consists of three main parts: the backbone, the neck, and the head. The output of the head generates feature maps with sizes of 160 × 160, 80 × 80, 40 × 40, and 20 × 20.

YOLOv8’s backbone uses the CSPDarknet53 architecture, which incorporates multiple C2f modules and an SPPF module to enhance the receptive field. As feature extraction largely depends on the C2f module, enhancements were made to improve its capability. Furthermore, a new SPPF module was introduced to further expand the receptive field. The neck of YOLOv8 retains the Feature Pyramid Network (FPN) and Path Aggregation Network (PAN) for feature fusion, with improvements for better integration of shallow and deep information. The head, originally composed of multiple convolutional modules, was enhanced with dynamic convolution to improve detection. These improvements culminate in the proposed MSGD-YOLO, as shown in Fig. 1.

The MSGD-YOLO network structure introduces GD-C2f and SPPELAN modules to reconstruct the backbone. The FPN and PAN structures in the neck are replaced by the more efficient MSGFPN to improve multiscale feature fusion. Finally, DyHead replaces the original detection head for improved detection performance.

### Reconfigured backbone network

Conventional CNNs typically require many parameters and floating-point operations (FLOPs) to achieve satisfactory accuracy. Given the significant redundancy in intermediate feature maps produced by mainstream CNNs, the Ghost module, a convolution module proposed by GhostNet^46^, has been introduced. This module employs more efficient linear operations to generate additional feature maps, achieving the same results as traditional convolution. This building block generates more feature maps with fewer parameters and computations. Each convolution operation that generates n feature maps from input data $X\in\:{R}^{C\times\:h\times\:w}$ with *C* input channels is expressed in Eq. (1), where *h* and *w* are the height and width of the input data, respectively.

1 $$\:Y=X\times\:w+b$$

Here, $\:Y\in\:{R}^{n\times\:{h}^{{\prime\:}}\times\:{w}^{{\prime\:}}}$ represents the output feature map, while $\:w\in\:{R}^{C\times\:k\times\:k\times\:n}$ represents the convolution kernels, and *b* is the bias term. The FLOPs required for this convolution are calculated as $\:n{\cdot\:h}^{{\prime\:}}\cdot{w}^{{\prime\:}}\cdot C\cdot\:k\cdot\:k$. This value typically reaches hundreds of thousands, as the number of convolution kernels *n* and the number of channels *C* are often very large.

According to Eq. (1), the input and output dimensions clearly determine the number of parameters to be optimized. The Ghost module suggests that feature maps generated by mainstream CNN operations contain a large amount of redundancy, with many being similar to each other. These redundant feature maps can be generated individually using more efficient operations. The feature extraction process of the Ghost module to generate *m* feature maps $\:{Y}^{{\prime\:}}\in\:{R}^{n\times\:{h}^{{\prime\:}}\times\:{w}^{{\prime\:}}}$ can be expressed as Eq. (2):

2 $$\:{Y}^{{\prime\:}}=X\times\:{w}^{{\prime\:}}$$

where $\:{w}^{{\prime\:}}\in\:{R}^{C\times\:k\times\:k\times\:m}$ represents the filters, with $\:m\le\:n$. Other hyperparameters, such as kernel size, stride, and padding, remain consistent with ordinary convolution to ensure the same output feature map size. Linear operations are applied to generate repeating features, as shown in the following Eq. (3):

3 $$\:{Y}_{ij}=\:{{\Phi\:}}_{ij}\left({Y}_{i}^{{\prime\:}}\right),\:\forall\:i\:=\:1,\:\dots\:,\:m,\:j\:=\:1,\:\dots\:,\:s$$

Where $\:{Y}_{i}^{{\prime\:}}$ represents the *i*-th feature map in $\:{Y}^{{\prime\:}}$, and $\:{{\Phi\:}}_{ij}$ is the *j*-th linear operation for each $\:{Y}_{i}^{{\prime\:}}$​ to generate the *j*-th Ghost feature map $\:{Y}_{ij}$. The final feature map is obtained by splicing the feature map generated by the Ghost module with that from the original convolution. After applying the Ghost module, set the linear convolution kernel size to $\:d\times\:d$. By comparing the computation cost between the Ghost module and standard convolution, the theoretical improvement can be derived. As shown in Eq. (4).

4 $${C}_{S}=\frac{c\cdot\:k\cdot\:k\cdot\:{n\cdot\:w}^{{\prime\:}}{\cdot\:h}^{{\prime\:}}}{{c\cdot\:w}^{{\prime\:}}{\cdot\:h}^{{\prime\:}}\cdot\:k\cdot\:k+{(s-1)\cdot\:w}^{{\prime\:}}{\cdot\:h}^{{\prime\:}}\cdot\:d\cdot\:d}\approx\:\frac{s\cdot\:c}{s+c-1}\approx\:s$$

Where $\:d\times\:d$ approximates $\:k\times\:k$ in size, and $\:s\ll\:c$. Thus, the computation cost of the Ghost module is quantitatively $\:\frac{1}{s}$ of that of standard convolution. The parameter calculation follows a similar approach and can also be simplified to *s*.

**Fig. 2:**
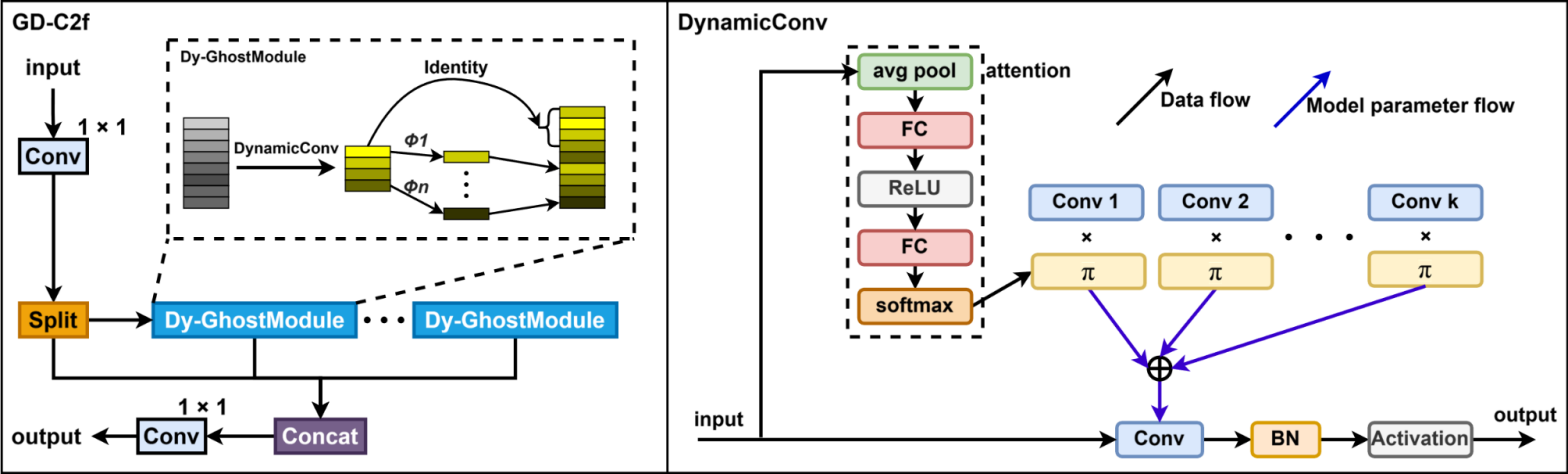
In the GD-C2f structure, the Ghost module replaces the original bottleneck structure, and dynamic convolution is introduced to replace standard convolutional layers within the Ghost module.

Leveraging the lightweight nature of the Ghost module, we integrated it into C2f to generate ghost feature maps, replacing some standard convolutions. However, ghost feature maps lack diversity, and the fixed convolution kernel fails to capture dynamic input features. Dynamic convolution replaces the standard operations in the Ghost module, enhancing feature diversity and adaptability. The final GD-C2f structure is shown in Fig. 2.

First, the standard convolution in the C2f module’s branch processing is replaced by Ghost module. Ghost module consists of two stages: preliminary feature generation and lightweight operations. In the preliminary feature generation stage, dynamic convolution replaces traditional convolution, dynamically selecting the most suitable kernel based on input features via a routing network, enhancing the flexibility and adaptability of feature extraction. In the lightweight operation stage of the Ghost module, dynamic convolution replaces the simple linear operation, further enhancing the accuracy and efficiency of feature generation. The branching structure and feature splicing mechanism of the C2f module are retained, fusing features processed by the Ghost module with skipped features, with final results output through 1 × 1 convolution. This improvement not only reduces computation and parameter cost but also enhances dynamic adaptability and feature expression.

**Fig. 3:**
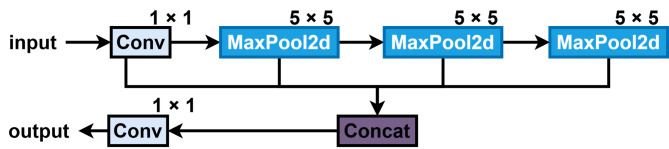
Structure of SPPELAN.

YOLOv8 originally employed the SPPF module for spatial pyramid pooling, enhancing the receptive field and capturing multi-scale features. However, it has limitations, such as suboptimal feature fusion. Replacing SPPF with the simpler and more efficient SPPELAN module enhances multi-scale feature extraction. The architecture of SPPELAN is shown in Fig. 3.

SPPELAN is an enhanced Spatial Pyramid Pooling-based architecture consisting of a convolutional module and three MaxPool modules for pooling. SPPELAN employs multiple MaxPools with a 5 × 5 kernel size to extract feature information at different scales. This enables a richer combination of receptive fields, capturing global context and target features more effectively at different scales. In contrast, SPPF uses only a single kernel size for multiple blending operations and has a relatively limited diversity of receptive fields. Additionally, SPPELAN’s tandem structure facilitates smoother gradient flow through the network. Each MaxPool module acts as a separate layer, allowing gradients to backpropagate through multiple paths, effectively mitigating vanishing or exploding gradients.

### Neck with multi-scale feature fusion

In the neck section of YOLOv8, FPN and PAN fuse multiscale feature maps to enhance feature representation and detection performance. However, this fixed fusion approach lacks adaptability. Thus, MSGFPN was redesigned based on the flexible and efficient fusion strategy of the Generalized Feature Pyramid Network (GFPN)^47^.

**Fig. 4:**
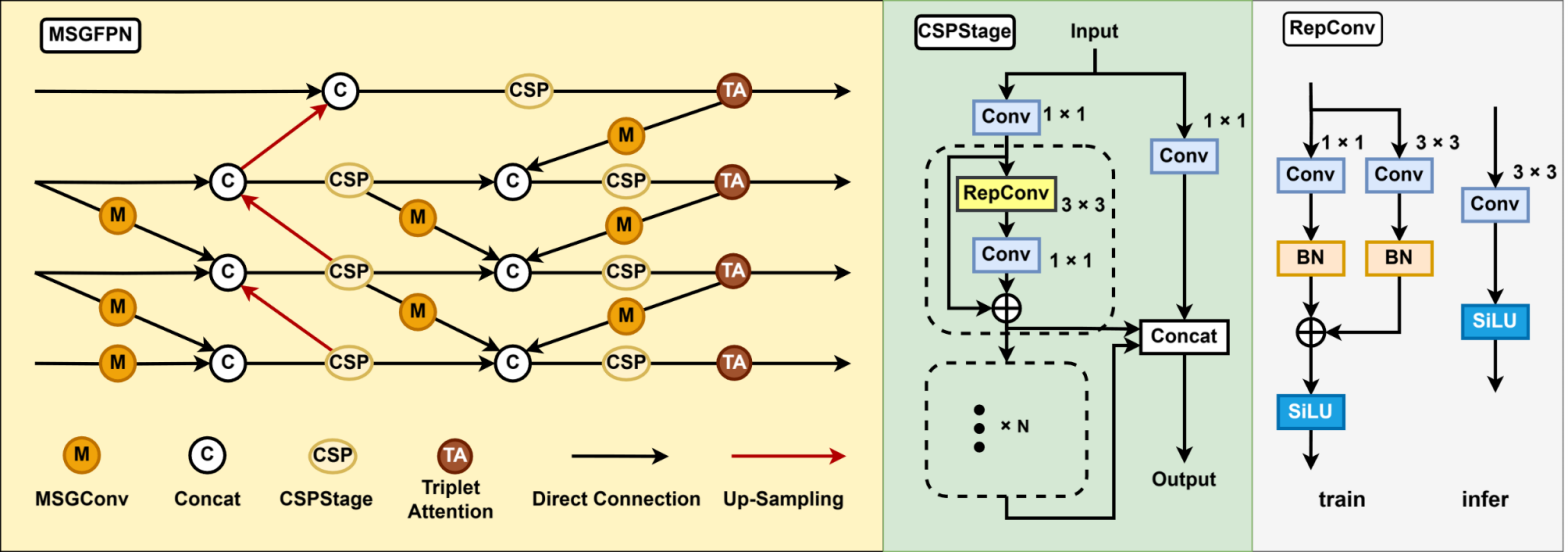
Structure of MSGFPN.

The structure of MSGFPN is shown in Fig. 4. During feature extraction, FPN performs multiple downsampling operations, leading to significant information loss during feature fusion. To mitigate the loss or blurring of small-target features, a small-target detection layer is added to GFPN. In MSGFPN’s feature transfer, MSGConv replaces ordinary convolution, reducing the computation and enhancing multi-scale feature extraction. CSPStage, derived from the Cross Stage Partial Network (CSPNet)^48^, uses RepConv instead of ordinary convolution to improve inference speed, and CSPStage replaces the 3 × 3 convolution-based feature fusion module in the original GFPN. During training, RepConv uses multiple branches to extract features of different scales and characteristics, while in inference, it merges these branches into a single equivalent convolutional layer through reparameterisation. This reparameterisation process significantly improves inference speed while reducing hardware demands without sacrificing model accuracy. Triple Attention^39^ is applied at the end of each information transfer branch to enhance the extraction of small-target information before sending the features to the network head.

**Fig. 5:**
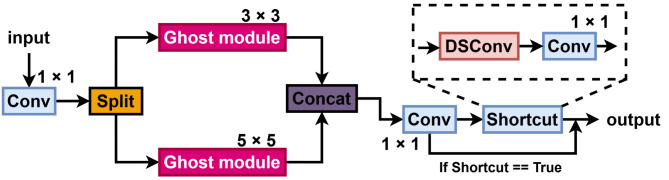
Structure of MSGConv. This module uses multi-scale concepts to enhance feature extraction at various scales.

Figure 5 illustrates the structure of the MSGConv proposed in this paper. The process begins with a 1 × 1 convolution applied to the input features, then the data are split and enter 3 × 3 and 5 × 5 Ghost modules, and finally merged. To prevent gradient vanishing and explosion, a residual block design is adopted, comprising DSConv and a convolution module to form the residual module.

**Fig. 6:**
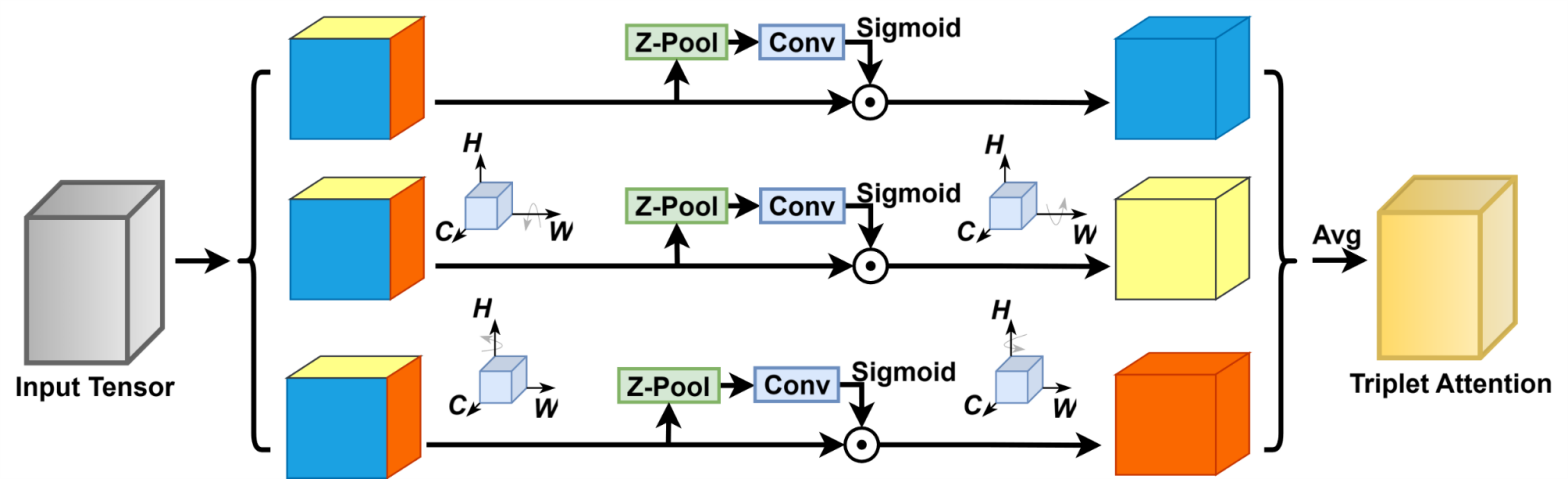
Structure of Triplet Attention. The attention consists of three branches that calculate the weights of the different dimensions of the features.

As shown in Fig. 6, the Triplet Attention^49^ enhances the neck’s performance in handling complex feature interactions. The bottom branch computes attention weights between the channel (C) and width (W). The middle branch calculates the weights between the channel (C) and height (H) dimensions, while the top branch computes the spatial correlation between height (H) and width (W). In the first two branches, a rotation operation connects the channel to the spatial dimensions, and the weights are averaged. The Z-pool reduces the tensor in the C to two dimensions, represented as shown in Eq. (5):

5 $$\:Z-pool\left(\alpha\:\right)=\left[MaxPoo{l}_{0d}\left(\alpha\:\right),\:\:AvgPoo{l}_{0d}\left(\alpha\:\right)\right]$$

The inclusion of Triplet Attention can capture the complex relationships between features more comprehensively, thus improving the feature representation capability of the model.

### DyHead

**Fig. 7:**
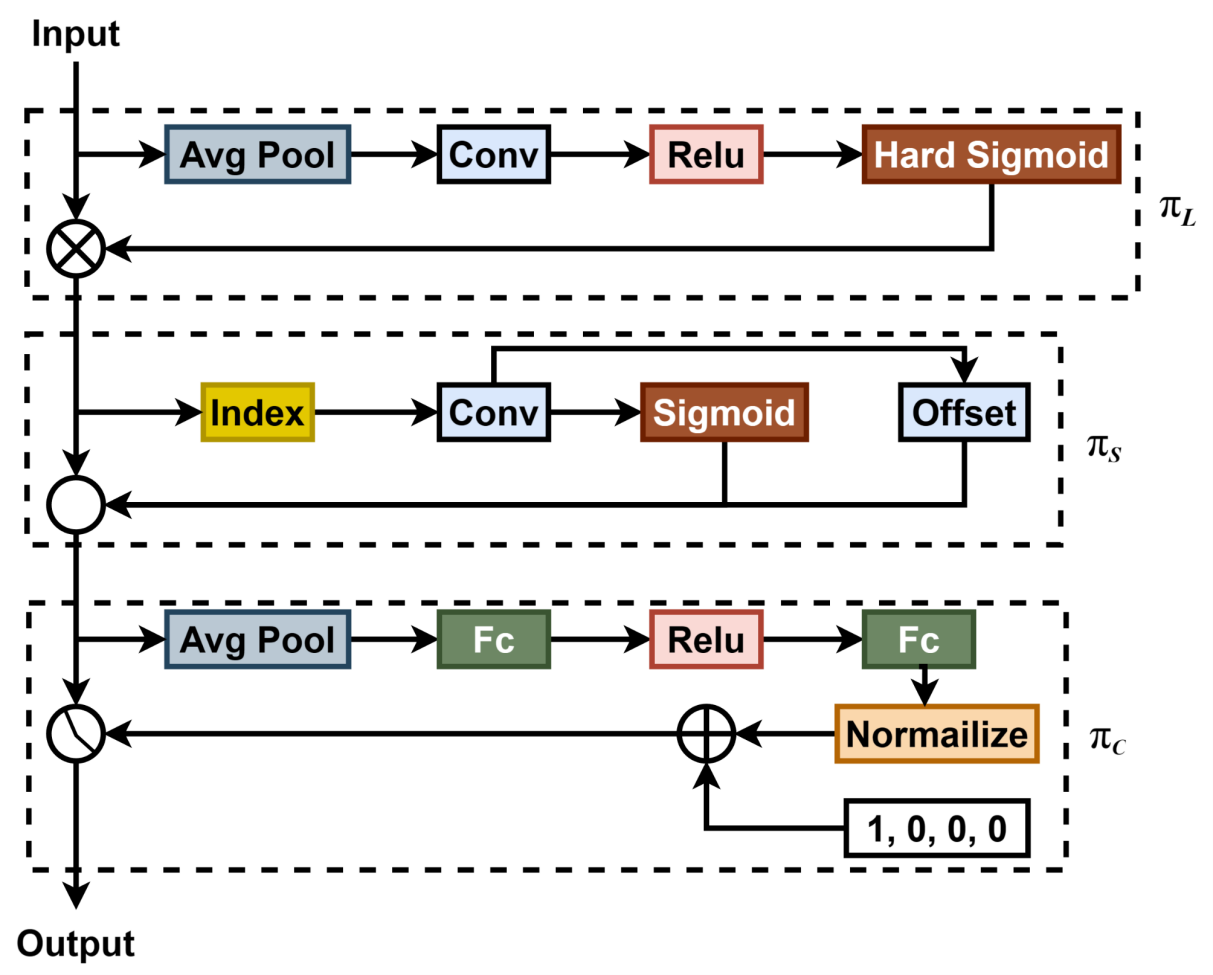
Structure of a DyHead. It consists of 3 attention modules, improved using a multi-step fusion.

Although YOLO’s traditional detection head is simple and fast, it has limitations in specific detection tasks. In contrast, DyHead enhances detection precision for small targets. By incorporating three self-attention mechanisms into the detection head, DyHead redefines the four-dimensional tensor $\:L\times\:H\times\:W\times\:C$ as a three-dimensional tensor $\:L\times\:S\times\:C$. This approach applies scale-aware, space-aware, and task-aware attention in the $\:L$, $\:S$, and $\:C$ dimensions, respectively. The structure of DyHead is illustrated in Fig. 7, where $\:{\pi\:}_{L}$, $\:{\pi\:}_{S}$, and $\:{\pi\:}_{C}$ correspond to the scale-aware attention, space-aware attention, and task-aware attention modules, respectively.

By leveraging attention mechanisms across feature hierarchies for scale perception, spatial locations for spatial perception, and output channels for task perception, this detection head significantly enhances the model’s expressive power without increasing computational complexity.

## Experiments and analysis

This section presents the implementation, experimental procedure, and results of the MSGD-YOLO network. The small target detection dataset (VisDrone2019) used for the experiments in this paper is presented, and based on performance results from the VisDrone2019 dataset, we demonstrate the superiority of the proposed model over YOLOv8-n and other benchmark models. Additionally, ablation experiments were conducted to verify the model’s validity.

### Dataset introduction

Due to our focus on small target detection, the VisDrone2019 dataset was selected for model training and testing. This dataset was collected by the AISKYEYE team at the Machine Learning and Data Mining Laboratory of Tianjin University and includes 8,599 still images across 10 categories. It includes numerous small targets (less than 32 × 32 pixels) and features diverse scenes and target scales.

**Fig. 8:**
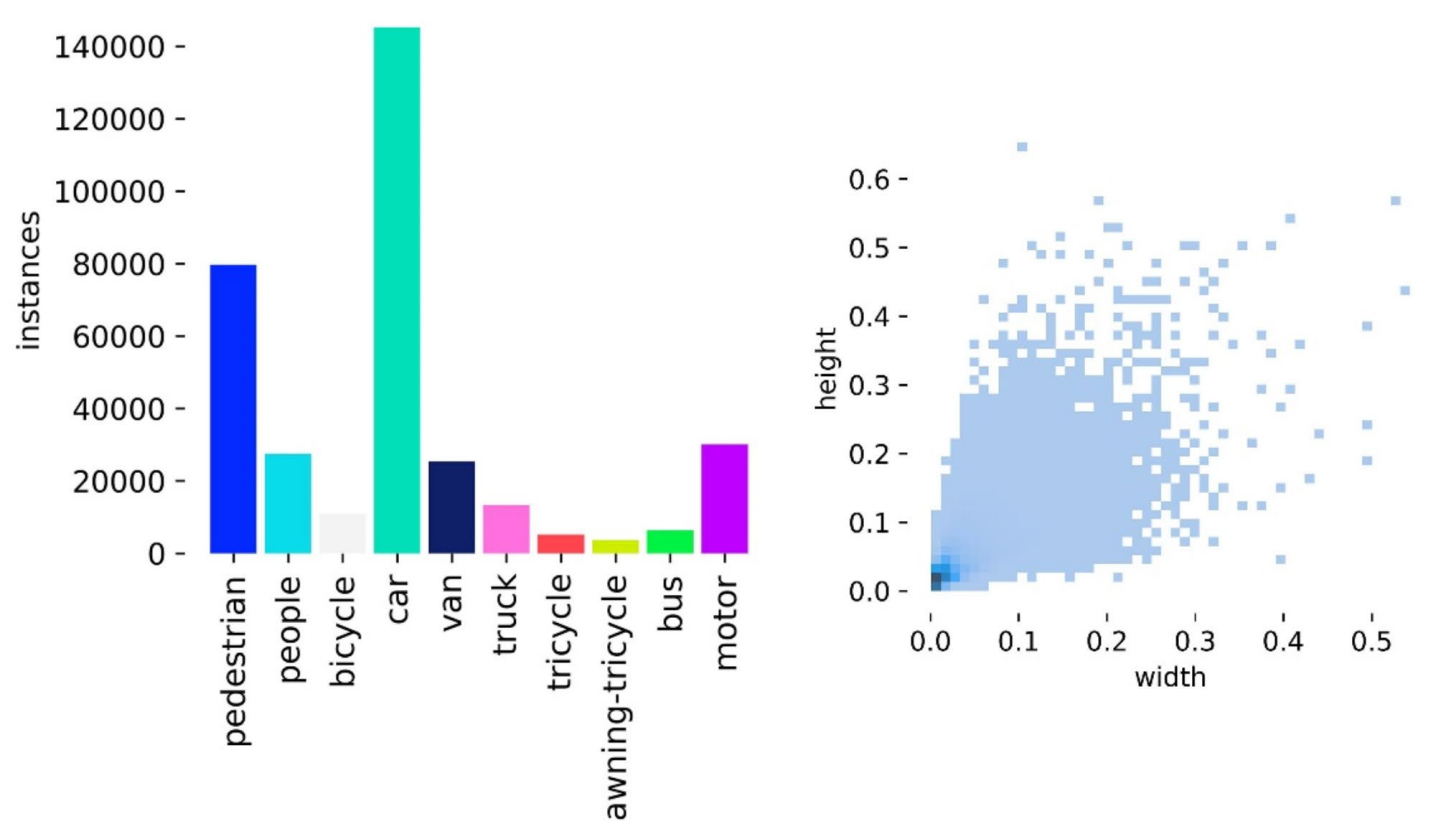
Number of species and instances and scale size in the VisDrone2019 dataset.

In Fig. 8, the dataset contains 10 common target objects, addressing daily detection needs. In the right figure, the target occupies a small portion of the image, aligning with small target detection requirements. From previous work, the VisDrone2019 dataset presents three key detection challenges: significant changes in object proportions, dense small objects, and blurred targets in low-light environments. These challenges demand high robustness in detection models and precision in small target recognition.

## Experimental setup and evaluation metrics

The experiment was conducted on a Windows 10 system with the following environment: Python version 3.11.2, PyTorch version 2.0.1, and CUDA version 12.4. All networks were trained on an NVIDIA GeForce RTX 3090 GPU (24 GB). The specific hyperparameter settings are detailed in Table 1.

**Table 1:** Training setup of the network.

Hyperparameter	Value
Epochs	300
Optimizer	SGD
Learning rate	0.01
Image size	640
Batch size	16
Momentum	0.937

In this experiment, the performance of the MSGD-YOLO model was evaluated via the following metrics: Precision (P), Recall (R), Mean Average Precision (mAP), number of parameters (Params), Giga Floating Point of Operations (GFLOPs), and FPS. The formulas for P, R, mAP, and mAP@50–95 are shown in Eqs. (6), (7), (8) and (9):

6 $$\:Precision=\frac{TP}{TP+FP}$$

7 $$\:Recall=\frac{TP}{TP+FN}$$

8 $$\:mAP=\frac{1}{N}\sum_{i=1}^{N}A{P}_{i}$$

9 $$\:mAP@50-95=\frac{1}{N}\sum_{j=1}^{N}mA{P}_{IoU=0.5+0.05\left(j-1\right)}$$

Among the metrics assessed, precision measures the proportion of predicted positives that are true positives. True Positives (TP) are correctly predicted positives, while False Positives (FP) are incorrectly predicted positives. Recall measures the proportion of actual positives correctly identified by the model. False Negatives (FN) are actual positives misclassified as negative by the model. The mAP@50 represents the mean precision at an Intersection over Union (IoU) threshold of 0.5, while the mAP@50–95 is the mean precision averaged over IoU thresholds from 0.5 to 0.95, in steps of 0.05.

The number of parameters represents the total weighting parameters in the model, used to evaluate its memory consumption. GFLOPs represent the number of floating-point operations during inference, estimating the model’s complexity. FPS is the number of images processed per second, indicating the model’s inference speed.

### Validity analyses and ablation experiments

In this section, we conduct several comparative experiments on the VisDrone2019 dataset and analyse the feasibility of the results. To validate the effectiveness of the proposed MSGFPN structure, a comparative experiment was conducted. In the baseline YOLOv8-n model, we replaced the neck with FPN^16^, BiFPN^50^, and NAS-FPN^17^ as the control group. The YOLOv8-n model with MSGFPN served as the experimental group. To ensure fairness, four detection heads were tested in the same experimental environment. The results after training on the same dataset are presented in Table 2.

**Table 2:** The performance of different feature pyramid networks and the MSGFPN on VisDrone 2019.

Method	mAP@50	mAP@50–90	Params(M)	GFLOPs
YOLOv8-n + FPN	35.1	19.3	3.01	15.8
YOLOv8-n + BiFPN	35.9	19.7	2.88	12.8
YOLOv8-n + NAS-FPN	37.5	20.3	2.80	13.2
YOLOv8-n + MSGFPN	40.5	23.4	2.77	12.5

As shown in Table 2, the proposed MSGFPN outperforms FPN, BiFPN, and NAS-FPN in mAP, GFLOPs, and Params. This demonstrates that MSGFPN is more suitable for small target detection than the other feature pyramid networks. Additionally, it is lighter and better suited for deployment on mobile-embedded devices.

Similarly, we explored the impact of varying numbers of DyHead blocks on the network. The results are shown in Table 3. Based on the results, we determined that the optimal number of DyHead blocks is 2.

 To further verify the effectiveness of the proposed enhancement strategy, we conducted an ablation experiment to explore the impact of different strategies on object detection performance. Under the same test conditions, YOLOv8-n was used as the benchmark model, and the proposed improvement strategies were gradually incorporated. No pre-trained weights were used during training to ensure fairness. The experimental results are presented in Table 4.

**Table 3:** Comparison of the number of different DyHead blocks.

Number	mAP@50	Params(M)	GFLOPs
1	32.7	3.01	9.4
2	34.0	3.32	10.7
3	34.8	4.06	12.1
4	35.1	4.87	14.7
5	35.5	5.73	16.1
6	35.8	6.89	18.5

**Table 4:** Ablation experiment.

GD-C2f	SPPELAN	MSGFPN	DyHead	mAP@50	Map@50–95	Params(M)
				31.1	16.3	3.11
✓				32.7	17.8	2.43
	✓			31.9	17.0	2.85
		✓		42.7	23.2	2.59
		✓	✓	43.1	24.1	2.83
✓	✓		✓	39.6	21.1	2.77
✓	✓	✓	✓	45.2	27.5	2.61

As shown in Table 4, integrating different enhancement strategies improves mAP and reduces the number of parameters compared to the baseline model. The ablation experiments lead to the following conclusions: (1) GD-C2f and SPPELAN effectively address the limited feature expression of C2f in the baseline model and reduce the model’s parameters. (2) Replacing the neck with MSGFPN improves detection precision and reduces parameters. (3) Dyhead enhances detection across scales and increases mAP.

**Fig. 9:**
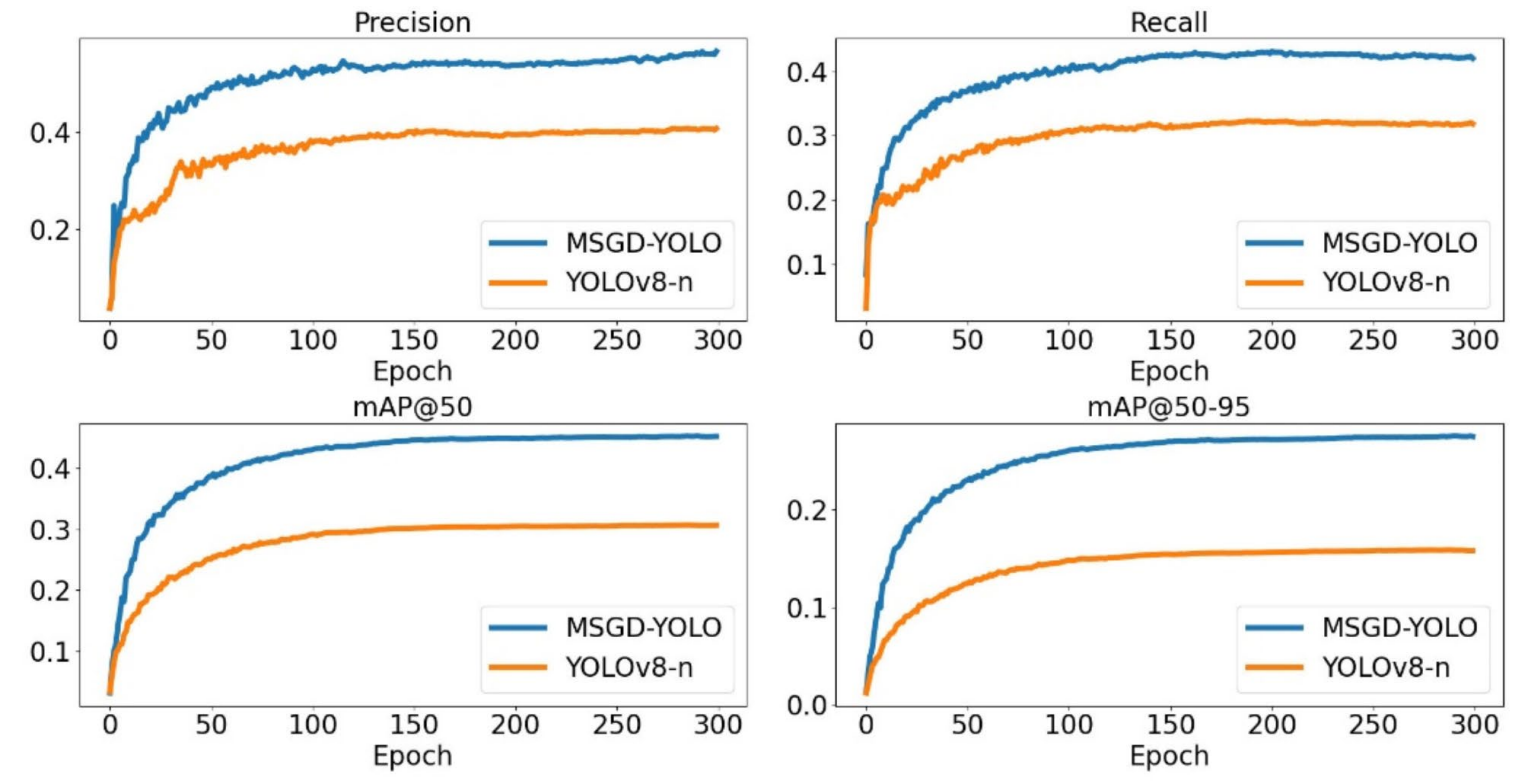
Diagram of the training process of MSGD-YOLO with YOLOv8-n.

Figure 9 shows that after the changes, MSGD-YOLO’s P, R, and mAP during training are significantly higher than those of the benchmark model, YOLOv8-n. MSGD-YOLO’s ability to achieve a higher mAP with fewer parameters than YOLOv8-n validates the effectiveness of our proposed method.

### Comparative experiments with other target detection algorithms

To verify the performance of MSGD-YOLO, we compared the proposed model with current mainstream YOLO models (including the latest state-of-the-art model, YOLOv10^51^) and variants of the YOLO model applied in the field of small target detection on the VisDrone2019 dataset. The comparison results are shown in Table 5.

**Table 5:** Performance comparison between MSGD-YOLO and other mainstream models.

Method	mAP@50	mAP@50–95	Params(M)	GFLOPs
Faster R-CNN^52^	31.0	17.2	41.2	118.8
YOLOv3-tiny^53^	15.8	6.9	8.7	12.9
YOLOv3	35.4	16.8	61.5	154.7
YOLOv5-n^54^	24.2	12.2	1.8	4.5
YOLOv5-s	30.6	15.6	7.2	15.8
YOLOv7-tiny^55^	25.2	11.8	6.0	13.1
YOLOv8-n^56^	31.1	16.3	3.1	8.7
YOLOv8-s	38.5	21.4	11.1	28.5
YOLOv10-n	32.1	18.3	2.7	8.4
YOLOv10-s	37.6	21.1	8.1	24.8
RT-DETR-R18^57^	44.1	25.1	20.1	30.4
RT-DETR-R34	45.1	26.8	31.1	91.8
BDH-YOLO^58^	42.9	26.2	9.4	-
MS-YOLO-s^59^	44.3	25.0	9.6	-
EM-YOLO^60^	43.0	25.1	8.9	25.6
MSGD-YOLO	45.2	27.5	2.6	21.5

As shown in Table 5, MSGD-YOLO achieves higher mAP@50 and mAP@50–95 than other mainstream YOLO models. It achieves the best performance across all evaluation metrics. MSGD-YOLO has only 2.6 MB of parameters, slightly larger than YOLOv5-n but significantly smaller than other models. In terms of GFLOPs, MSGD-YOLO achieves the highest mAP with fewer computations than other YOLO variants. These results demonstrate that MSGD-YOLO identifies small target objects more accurately and quickly, making MSGD-YOLO more suitable for embedded deployment.

### Performance on embedded devices

**Fig. 10:**
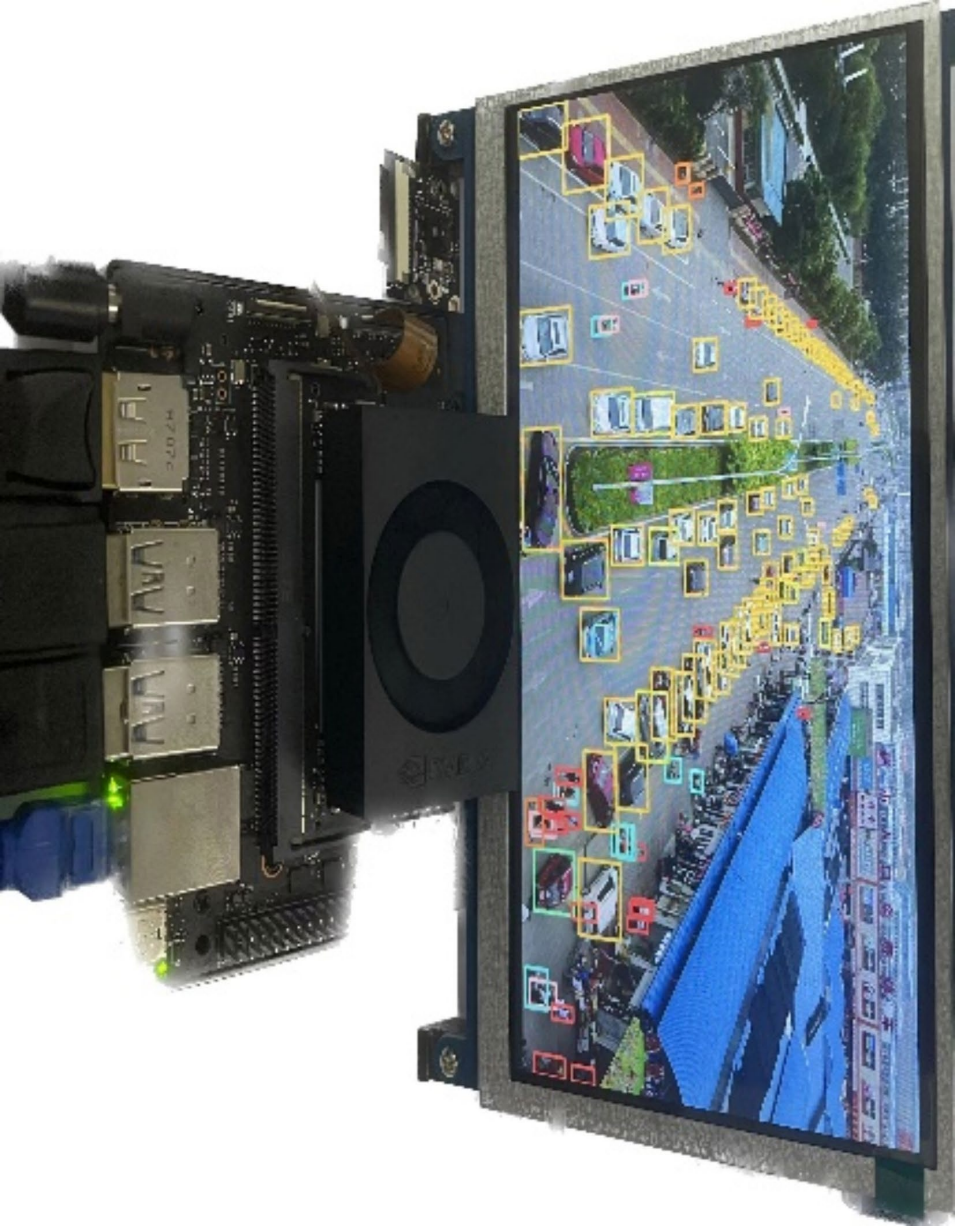
Actual pictures of the Jetson Orin Nano.

To compare the performance of the proposed method with other lightweight object detection algorithms, inference speed was tested on the Jetson Orin Nano, equipped with 16 tensor cores and 512 Nvidia ampere architecture GPU cores. The Jetson Orin Nano is shown in Fig. 10.

The FPS of various lightweight models, including MSGD-YOLO, was tested on this device, along with other metrics for a comprehensive comparison. The results are shown in Table 6.

**Table 6:** Performance of MSGD-YOLOv8n and other lightweight target detection models on the Jetson Orin Nano.

Method	mAP@50	Params(M)	FPS
YOLOv8-n	31.1	3.1	39.2
YOLOv8-s	38.5	11.1	19.3
YOLOv10-n	32.1	8.1	40.6
MSGD-YOLO	45.2	2.6	24.6

The table shows that although the FPS of MSGD-YOLO is reduced, it still meets the real-time detection requirements for practical applications. In scenarios requiring high-precision small target detection, MSGD-YOLO sacrifices some FPS compared to the baseline model but significantly improves accuracy. Thus, the proposed method achieves a good balance between real-time detection and accuracy.

### Visual analysis

**Fig. 11:**
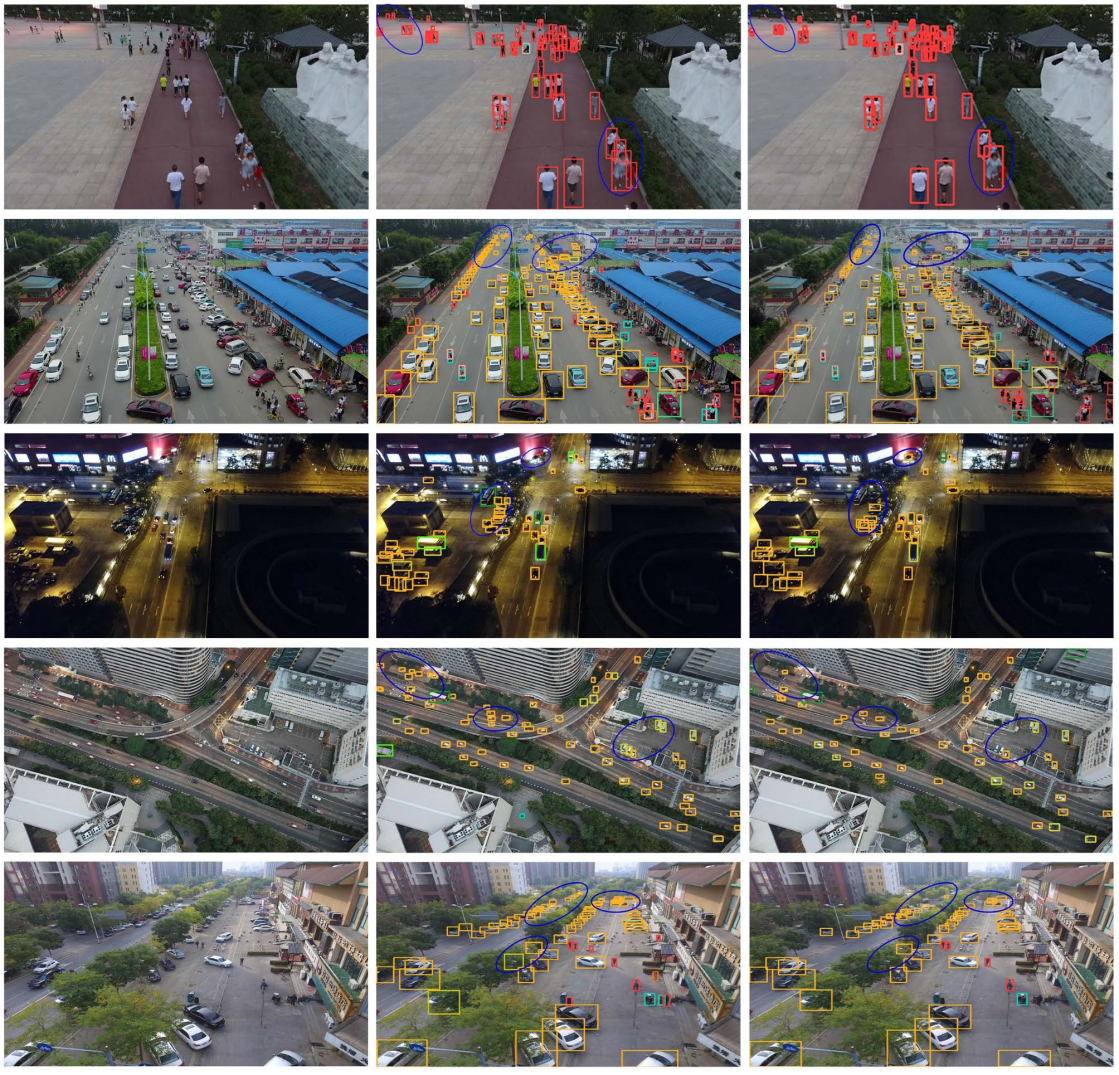
MSGD-YOLO (middle) and YOLOv8-n (right) detection results.

To demonstrate MSGD-YOLO’s detection performance across different scenes, images from five scenes were selected for detection and analysis. The scenes include an open field with dense pedestrians, a complex road, an intersection in low-light conditions, an overhead view of small targets, and a heavily occluded target detection scene. Objects in these images were detected using YOLOv8-n and MSGD-YOLO, and the detection results are shown in Fig. 11.

The detection results show that MSGD-YOLO achieves a significantly higher detection rate than the baseline model for distant tiny targets. Under low-light conditions, the proposed model continues to identify small targets accurately, with no false detections compared to YOLOv8-n. In the overhead view, the model maintains a high detection rate for small targets occupying very few pixels. In the heavily occluded scene, despite limited feature information, MSGD-YOLO successfully detects the targets. These results demonstrate that MSGD-YOLO exhibits strong robustness and a high detection rate.

**Fig. 12:**
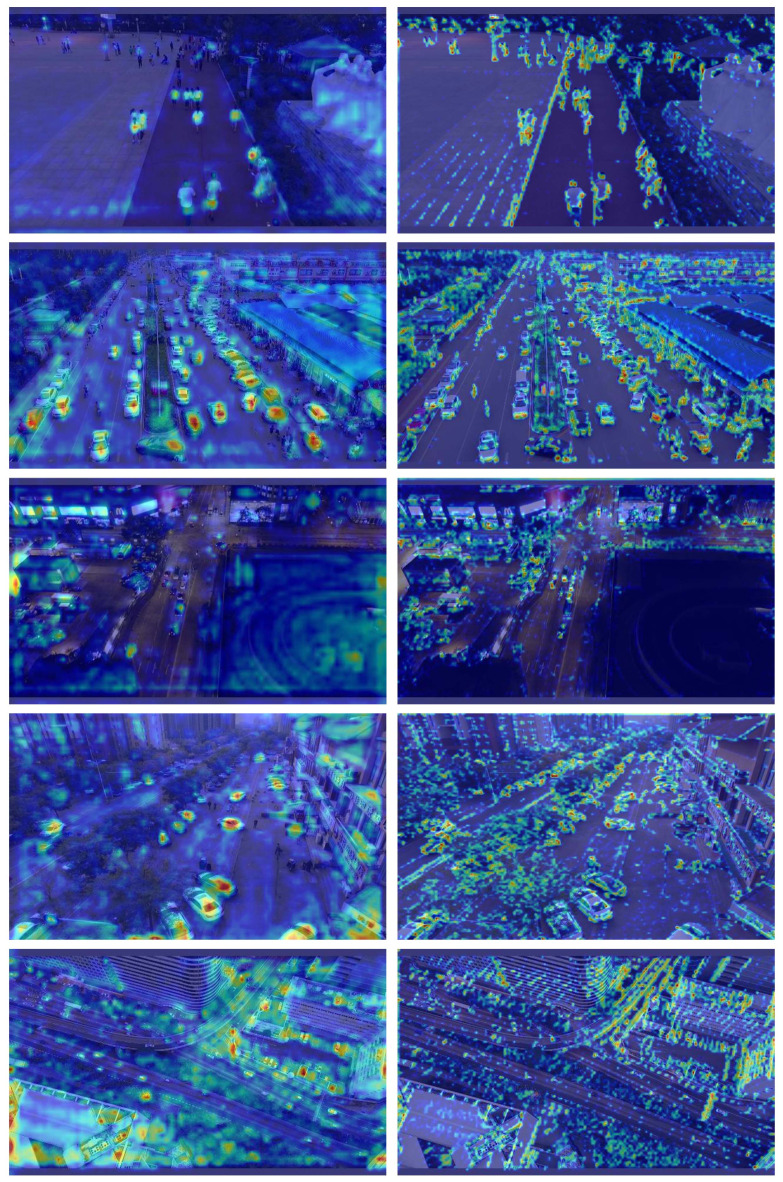
Heatmaps of MSGD-YOLO (right) and YOLOv8-n (left).

In Fig. 12, the heatmaps reflect the model’s regions of interest and localisation accuracy. The improved MSGD-YOLO exhibits superior feature extraction and localisation capabilities. This is evident in the concentrated and intensified high-response regions, which are highly noticeable. In contrast, the baseline model shows more dispersed highlights, unevenly distributed over the target area. As they do not fully cover the target, this causes localisation bias in small targets, reducing detection efficiency. Additionally, bright colours in the background hinder the model’s ability to distinguish targets from non-targets, increasing the false alarm rate. In the improved MSGD-YOLO heat map, bright-coloured regions of the target area have clear boundaries, aligning with the actual contours of the targets. No overlap occurs between the highlighted regions of different targets, indicating that the model accurately locates and separates multiple targets. As a result, MSGD-YOLO is more capable of detecting small targets than the baseline model.

## Conclusions

To detect small objects with limited computing and storage resources on embedded devices accurately, the structure of each part of the YOLOv8-n network was analysed and redesigned. An MSGD-YOLO network for small target detection on embedded devices is subsequently proposed.

The MSGD-YOLO network was tested on the VisDrone2019 dataset. Compared with the YOLOv8-n network, MSGD-YOLO improves target detection accuracy and reduces the number of parameters. Compared with other lightweight networks, the MSGD-YOLO network achieves the best detection accuracy with the second smallest number of parameters and a moderate amount of computation. Compared with larger networks, MSGD-YOLO achieves the highest detection accuracy with the fewest parameters and computations. Furthermore, compared with other lightweight models on embedded devices, it meets the requirements for real-time detection. These results verify the effectiveness of the proposed network.

Currently, MSGD-YOLO has several limitations and has only been tested on the VisDrone2019 dataset. The algorithm needs further optimization to ensure real-time detection on embedded devices with ultralow computing power. Further research is needed to enhance its effectiveness in practical applications.

## Data Availability

The experimental test images in this paper are from the open source VisDrone dataset. (https://github.com/VisDrone/VisDrone-Dataset).
